# Supplementary figures and images for: The small GTPase ARF3 controls invasion modality and metastasis by regulating N-cadherin levels
Source: J Cell Biol. 2023 Feb 28;222(4):e202206115. doi: 10.1083/jcb.202206115 (PMC9997661; doi:10.1083/jcb.202206115)

Figure 3F

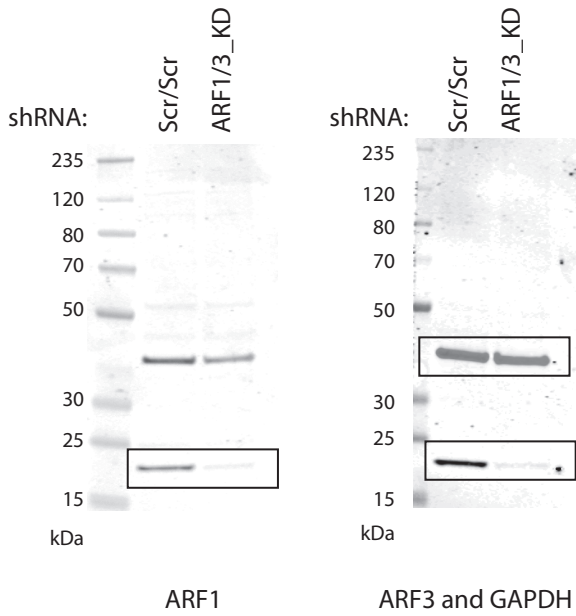

Supplement: SourceData F3 — is the source file for Fig. 3. [file JCB_202206115_SourceDataF3.pdf]

### Figure 4H

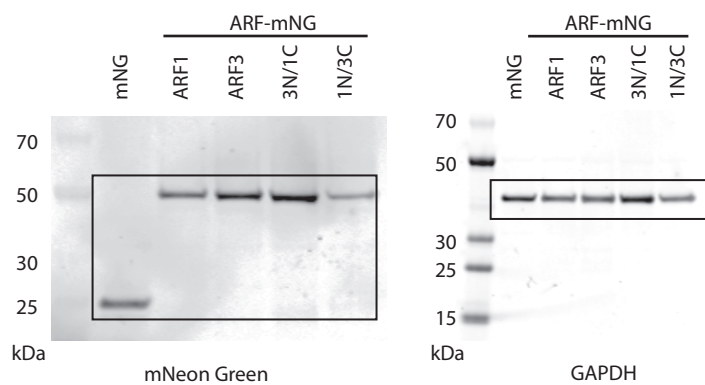

### Figure 4I

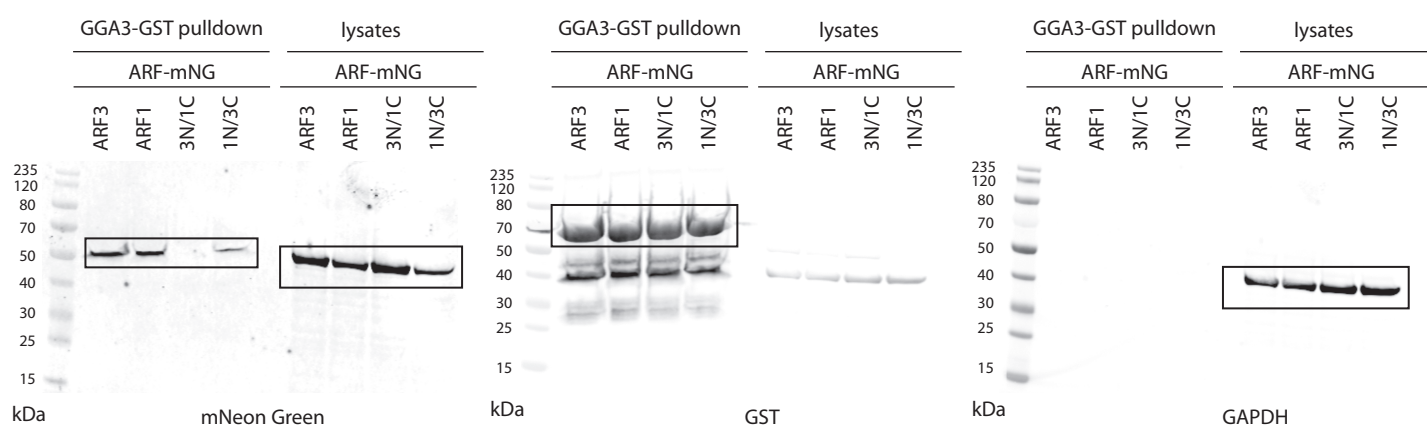

Supplement: SourceData F4 — is the source file for Fig. 4. [file JCB_202206115_SourceDataF4.pdf]

Figure 5A

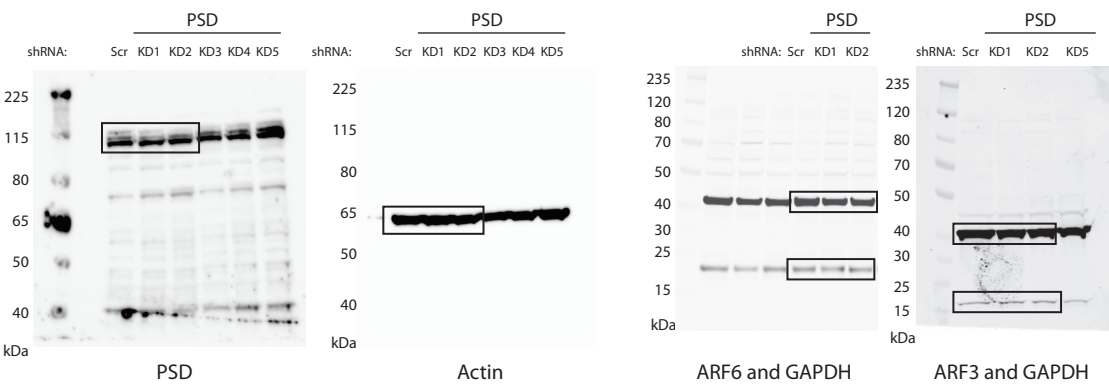

Figure 5B

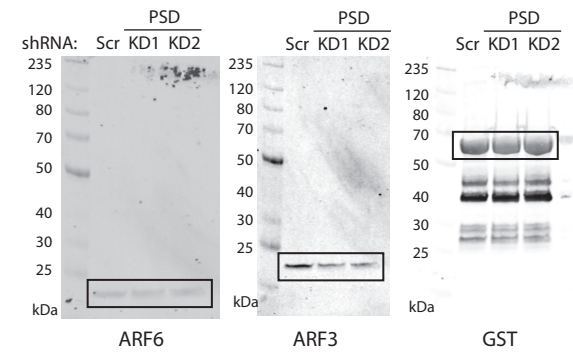

Figure 5F

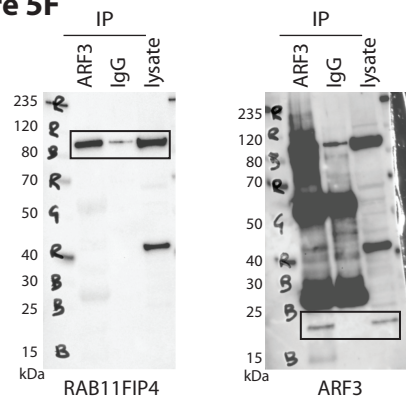

Figure 5G

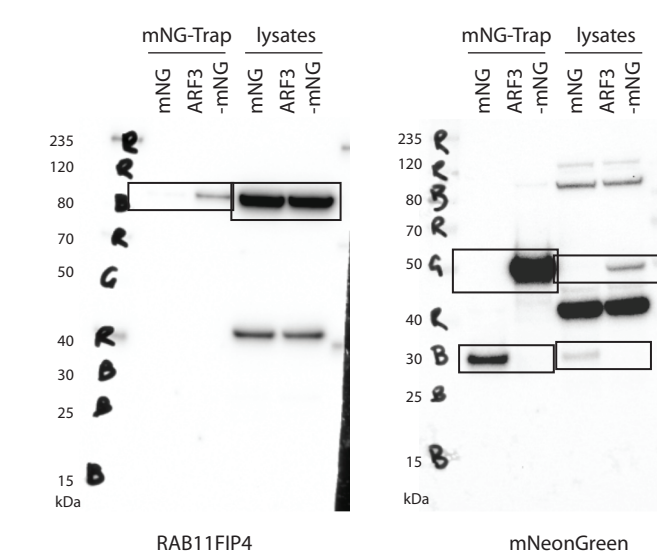

Figure 5K

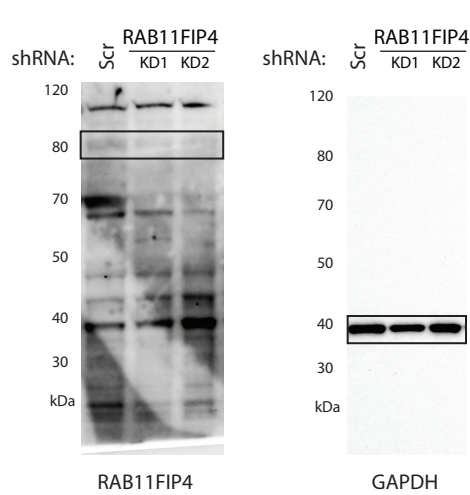

Supplement: SourceData F5 — is the source file for Fig. 5. [file JCB_202206115_SourceDataF5.pdf]

Figure 6B

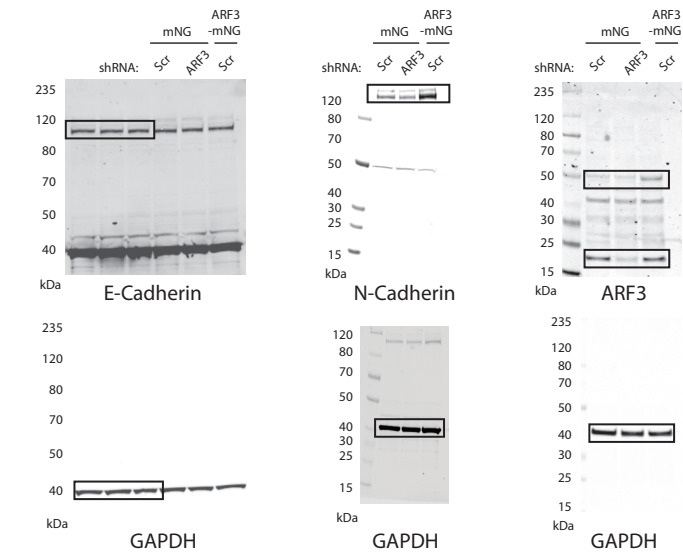

Figure 6F

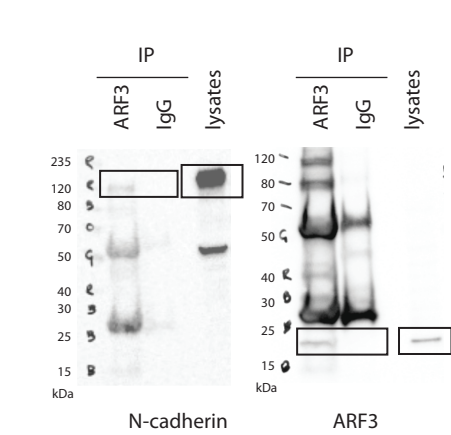

Figure 6O

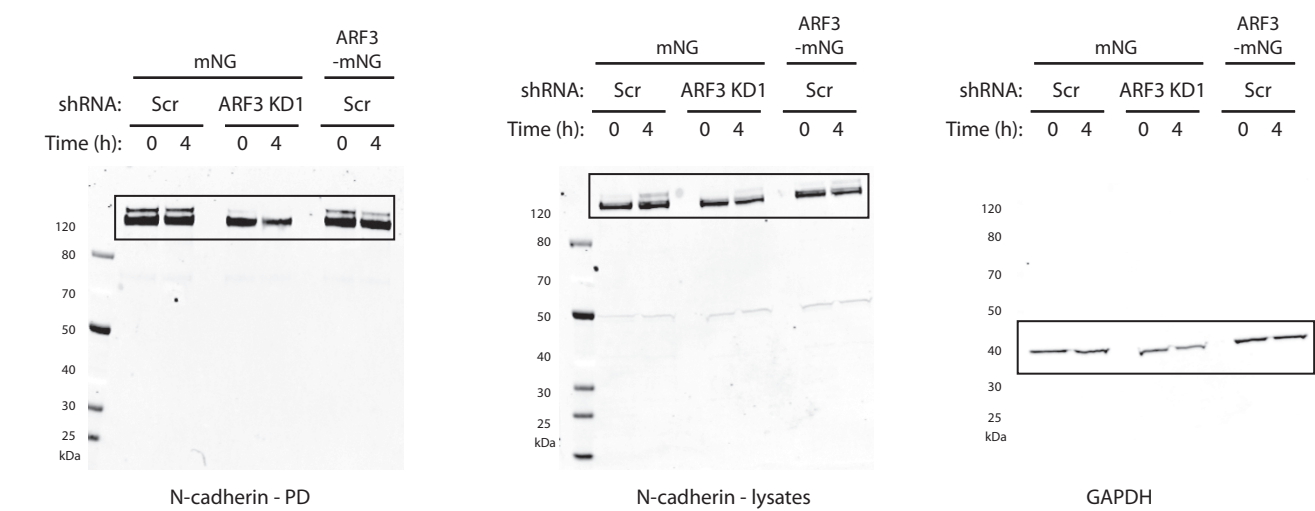

Supplement: SourceData F6 — is the source file for Fig. 6. [file JCB_202206115_SourceDataF6.pdf]

Supplementary Figure 4C

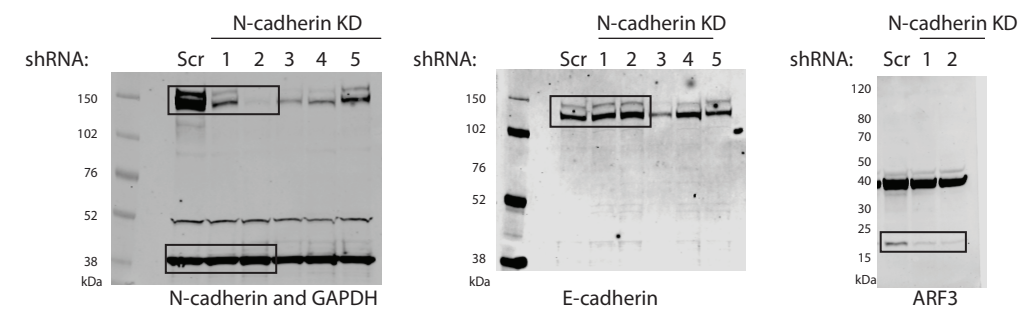

Supplementary Figure 4D

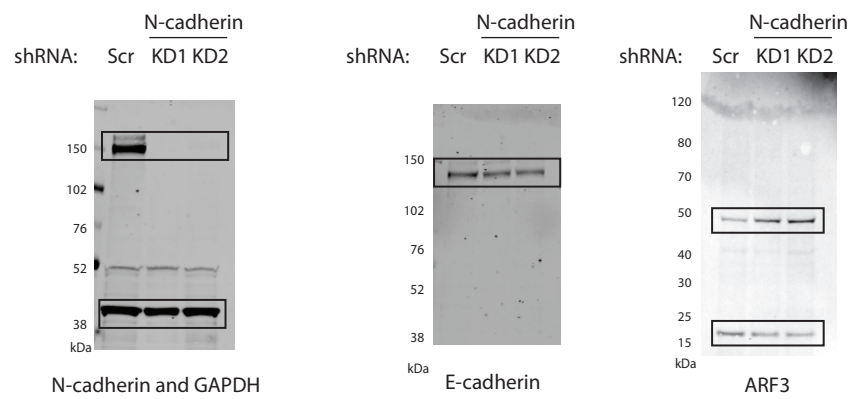

Supplementary Figure 4J

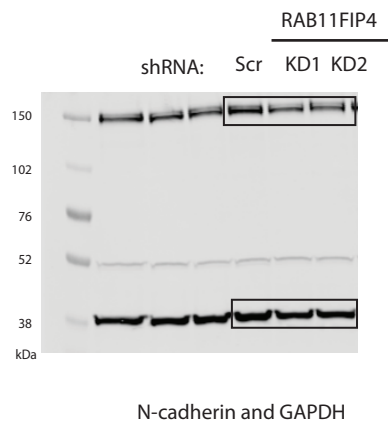

Supplement: SourceData FS4 — is the source file for Fig. S4. [file JCB_202206115_SourceDataFS4.pdf]
