# Supplementary material for: The small GTPase ARF3 controls invasion modality and metastasis by regulating N-cadherin levels
Source: J Cell Biol. 2023 Feb 28;222(4):e202206115. doi: 10.1083/jcb.202206115 (PMC9997661; doi:10.1083/jcb.202206115)

Supplementary Figure 1F

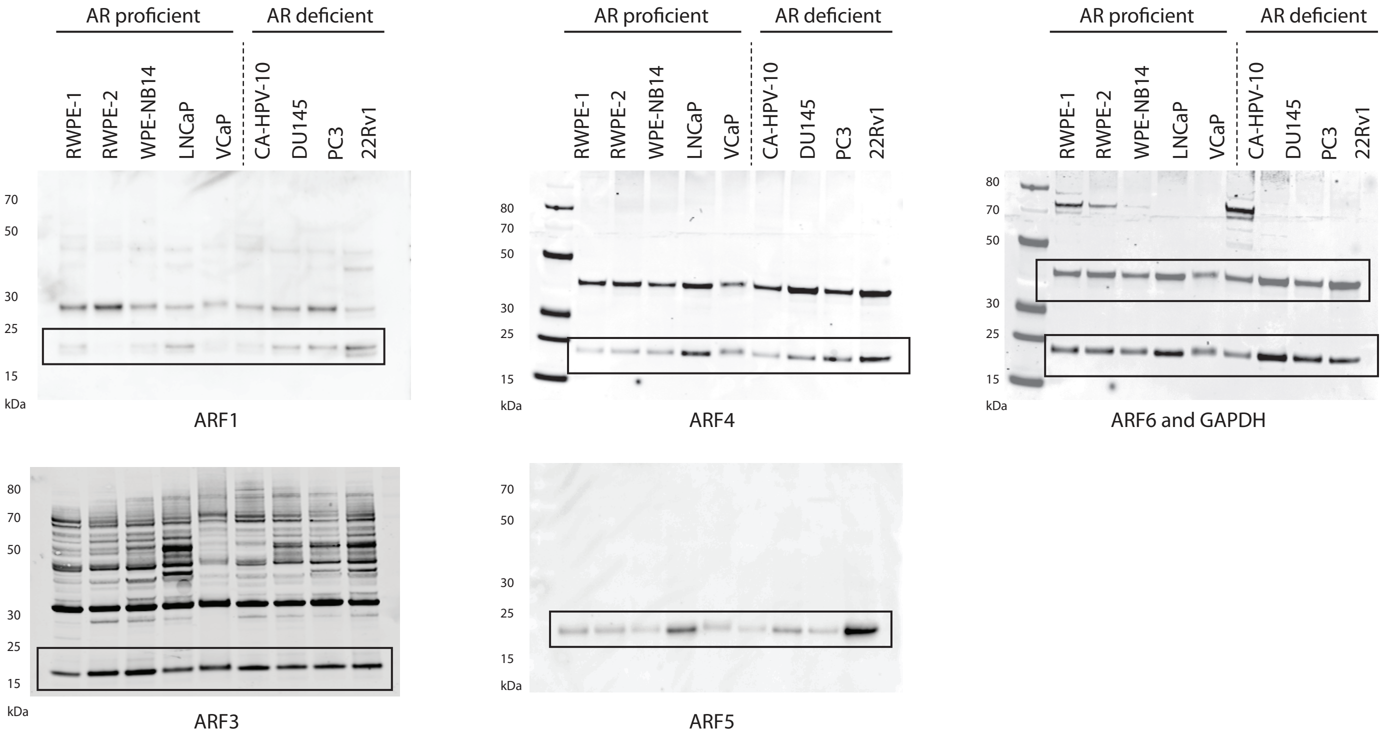

Supplementary Figure 1G

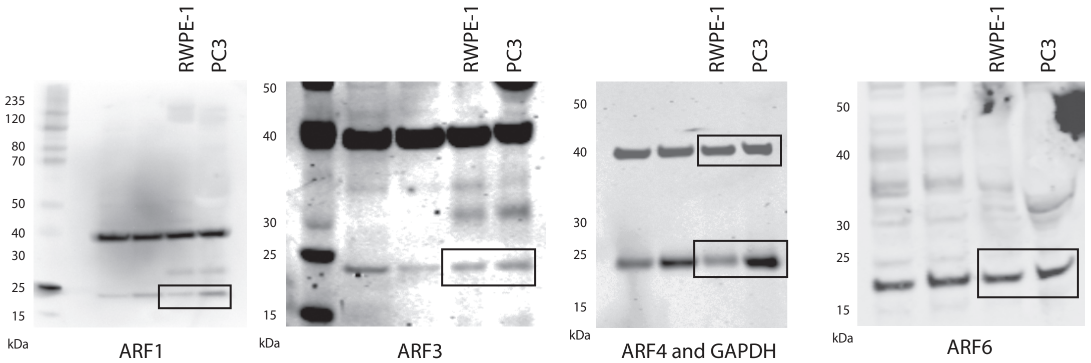

Supplementary Figure H-I

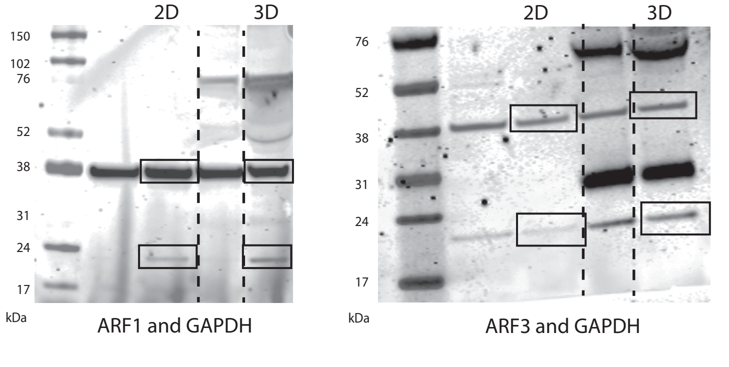

Supplementary Figure L-M

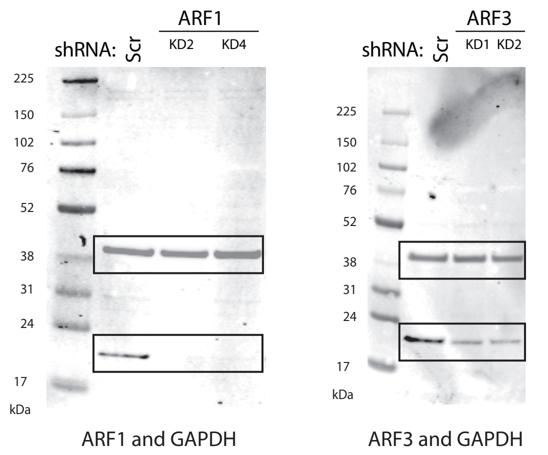

Supplement: SourceData FS1 — is the source file for Fig. S1. [file JCB_202206115_SourceDataFS1.pdf]
